# Differences in Ocular Complications Between *Candida albicans* and Non-albicans *Candida* Infection Analyzed by Epidemiology and a Mouse Ocular Candidiasis Model

**DOI:** 10.3389/fmicb.2018.02477

**Published:** 2018-10-17

**Authors:** Masahiro Abe, Yuki Kinjo, Keigo Ueno, Shogo Takatsuka, Shigeki Nakamura, Sho Ogura, Muneyoshi Kimura, Hideki Araoka, Sota Sadamoto, Minoru Shinozaki, Kazutoshi Shibuya, Akiko Yoneyama, Mitsuo Kaku, Yoshitsugu Miyazaki

**Affiliations:** ^1^Department of Chemotherapy and Mycoses, National Institute of Infectious Diseases, Tokyo, Japan; ^2^Department of Infection Control and Laboratory Diagnostics, Tohoku University School of Medicine, Miyagi, Japan; ^3^Department of Infectious Diseases, Toranomon Hospital, Tokyo, Japan; ^4^Department of Bacteriology, The Jikei University School of Medicine, Tokyo, Japan; ^5^Jikei Center for Biofilm Science and Technology, The Jikei University School of Medicine, Tokyo, Japan; ^6^Okinaka Memorial Institute for Medical Research, Tokyo, Japan; ^7^Department of Surgical Pathology, Toho University School of Medicine, Tokyo, Japan

**Keywords:** candidemia, ocular candidiasis, *Candida albicans*, neutrophils, monocytes, cytokines, chemokines

## Abstract

**Objectives:** *Candida* species are a major cause of hospital infections, including ocular candidiasis, but few studies have examined the propensities of specific species to invade the eye or the unique immunological responses induced. This study examined the frequency and characteristics of species-specific *Candida* eye infections by epidemiology and experiments using a mouse ocular candidiasis model.

**Methods:** We reviewed medical records of candidemia patients from January 2012 to March 2017. We also evaluated ocular fungal burden, inflammatory cytokine and chemokine profiles, and inflammatory cell profiles in mice infected with *Candida albicans*, *Candida glabrata*, or *Candida parapsilosis.*

**Results:** During the study period, 20 ocular candidiasis cases were diagnosed among 99 candidemia patients examined by ophthalmologists. Although *C*. *parapsilosis* was the most frequent candidemia pathogen, only *C*. *albicans* infection was significantly associated with ocular candidiasis by multivariate analysis. In mice, ocular fungal burden and inflammatory mediators were significantly higher during *C*. *albicans* infection, and histopathological analysis revealed invading *C*. *albicans* surrounded by inflammatory cells. Ocular neutrophil and inflammatory monocyte numbers were significantly greater during *C*. *albicans* infection.

**Conclusion:** *Candida albicans* is strongly associated with ocular candidiasis due to greater capacity for invasion, induction of inflammatory mediators, and recruitment of neutrophils and inflammatory monocytes.

## Introduction

*Candida* species are among the most common causative pathogens of bloodstream infections in hospitals, and ocular candidiasis is a potentially severe complication that can lead to visual field defects or blindness if appropriate therapy is delayed (Verfaillie et al., 1991; Wisplinghoff et al., 2004). Previous reports have shown that all *Candida* species can cause ocular complications and that ocular candidiasis occurs in approximately 10–25% of *Candida* infections (Donahue et al., 1994; Oude Lashof et al., 2011; Durand, 2017). Moreover, the guidelines of the Infectious Diseases Society of America (IDSA) recommend that the fundus of all candidemia patients be examined by ophthalmologists within 1 week of diagnosis (Pappas et al., 2016). Several clinical studies found that *Candida albicans* was the species most frequently causing ocular complications, whereas others reported that ocular inflammation was most often caused by *Candida parapsilosis* (Donahue et al., 1994; Oude Lashof et al., 2011; Ranjith et al., 2017). Therefore, the species with the greatest propensity for causing ocular candidiasis is still uncertain. According to the IDSA, neutrophils are thought to be the primary effectors of ocular inflammation during candidiasis. However, the detailed immunological responses to specific *Candida* species and the factors inducing ocular inflammation remain obscure (Pappas et al., 2016).

To address these issues, we retrospectively reviewed the epidemiology of in-hospital ocular candidiasis and compared fungal burdens, chemotactic factor profiles, and inflammatory cell profiles in eyes after infection by specific *Candida* species in a mouse model.

## Materials and Methods

### Epidemiology

#### Medical Record Review

All cases of candidemia occurring between January 2012 and March 2017 at Toranomon Hospital (Tokyo, Japan, 890 beds) were identified. Candidemia was defined by the isolation of any *Candida* species from one or more sets of blood cultures from patients with signs of infection. Breakthrough candidemia was defined as candidemia occurring in a patient who had received systemic antifungal therapy for over 7 days before the positive blood culture (Antoniadou et al., 2003; Abe et al., 2014).

Ocular examinations were performed by ophthalmologists. All patients with *Candida* bloodstream infection examined by ophthalmologists were included in this study. Candidemia cases were excluded from consideration if (1) ophthalmologists could not examine the fundus because of the poor general condition or (2) patients died before the blood cultures were confirmed positive. The medical records of each patient were also reviewed to identify the primary disease, the presence of central venous catheter insertion, neutrophil counts, usage of immunosuppressive agents, presence of pre-treatment antifungal agents, serum 1,3-beta-d-glucan (BDG) values, and 30 day all-cause mortality. Serum BDG was measured using the Wako turbidimetric assay (Wako Pure Chemical Industries, Tokyo, Japan) with positivity cutoff of 11 pg/mL (Moro et al., 2003; Kawazu et al., 2004). In the epidemiological investigation, an opt-out approach was used for obtaining consent. This investigation was approved by the Human Ethics Review Committee of Toranomon Hospital (Approved Number: 1546).

#### Isolation and Identification of *Candida* Species

Blood culture samples were processed using Bactec 9240 and Bacterc FX systems (Becton, Dickinson and Company, Sparks, MD, United States). All *Candida* species were isolated on Sabouraud dextrose agar (Nippon Becton Dickinson Company, Ltd., Japan) at 35°C and identified at the species level by a Vitek or Vitek 2 system (bioMérieux, Marcy l’Etoile, France) for all germ tube-negative *Candida* yeast in the Toranomon Hospital. The D1/D2 regions and/or internal transcribed spacer regions of the rRNA gene from *Candida* isolates were sequenced to provide further support for species identity by the National Institute of Infectious Diseases (White et al., 1990; O’Donnell, 1993). This research was approved by the Human Ethics Review Committee of the National Institute of Infectious Diseases (Approved Number: 841).

### Animal Studies

#### Mice

Female C57BL/6J mice, 7–9 weeks old, were purchased from Japan SLC, Inc. (Shizuoka, Japan) and maintained under specific pathogen-free conditions at the National Institute of Infectious Diseases in Japan. All experiments were reviewed and approved by the Animal Care and Use Committee of the National Institute of Infectious Diseases. Protocols were designed to minimize animal suffering and limit the numbers used for experiments (Approved Number: 116111).

#### Yeast Strains and Infection Models

Reference strains of *C. albicans* (SC5314), *Candida glabrata* (CBS138), and *C. parapsilosis* (ATCC22019), and clinically isolated strains of *C*. *albicans* (SF-30), *C*. *glabrata* (SF-31), and *C*. *parapsilosis* (TOR-1) were used for mouse candidiasis model studies. Clinical strains were isolated from blood cultures of candidemia patients. *C. albicans* and *C*. *parapsilosis* were grown at 30°C and *C*. *glabrata* at 37°C for 2 days on yeast extract peptone dextrose (YPD) agar. *C. albicans* and *C*. *parapsilosis* were then grown at 30°C and *C*. *glabrata* at 37°C in YPD broth for 18–24 h. After incubation, yeasts were collected, washed, and resuspended in sterile PBS at approximately 2.5–3.5 × 10^6^ colony-forming units (CFU)/mL. Mice were injected with 200 μL (approximately 5–7 × 10^5^ CFU/mouse) via the lateral tail vein to cause *Candida* dissemination.

#### Evaluation of Organ Fungal Burden and Cytokine Production

In this experiment, mice were divided into three groups as follows: *C. albicans*, *C. glabrata*, or *C. parapsilosis* infected group. The infected mice were euthanatized 3 days after injection, followed by aseptic collection of kidneys and eyeballs. The kidneys and eyeballs were homogenized in sterile PBS, and the homogenates of the kidneys were then serially diluted. Kidney homogenate was plated on YPD agar and eyeball homogenate on YPD agar with antibiotics (penicillin/streptomycin), both at 100 μL per plate. The fungal burden was determined by counting CFUs after 1 day incubation at 30°C. The remaining homogenates were centrifuged and stored at -30°C for cytokine analysis. Cytokines were measured by commercial enzyme-linked immunosorbent assay (ELISA) kits (interleukin [IL]-6, IL-12 p40, and tumor necrosis factor [TNF]-α kits from BD Biosciences, Franklin Lakes, NJ, United States; IL-1β, C-X-C motif ligand 1 [CXCL1]/KC, CXCL2/MIP-2, and C-C motif ligand 2 [CCL2]/MCP-1 kits from R&D Systems, Minneapolis, MN, Unites States).

#### Ocular Cell Isolation and Flow Cytometry

In this experiment, mice were divided into four groups as follows: uninfected control, *C. albicans*, *C. glabrata*, or *C. parapsilosis* infected group. The eyeballs were carefully enucleated and then washed with sterile PBS to eliminate contaminating peripheral blood. After washing, samples were incubated in RPMI medium with 2% fetal bovine serum and digestive enzymes (1 mg/mL hyaluronidase, Sigma-Aldrich, St. Louis, MO, United States; 2 mg/mL collagenase-D; and 10 μg/mL DNase I, Roche, Basel Switzerland) under rotation at 37°C for 1 h. After enzyme digestion, the eyeballs were homogenized and filtered to remove undigested particles. The ocular immune cells recovered in the filtrate were blocked with Fc-receptor blocking antibody and stained with fluorochrome-conjugated antibodies (Listed in the **Supplementary Table S1**). Immune cells were enumerated using the FACSCanto II^®^ flow cytometer (BD Biosciences). Obtained data were analyzed using FlowJo, version 9.5 (Tree Star, Inc., Ashland, OR, United States).

#### Histopathological Analysis of Infected Mouse Eyes

For histopathological analysis, eyeballs were removed 3 days after yeast injection and fixed in 10% formalin, dehydrated with ethanol, and embedded in paraffin following standard procedures. Five micrometer tissue sections were mounted onto glass slides (Dako, Japan) and stained with hematoxylin and eosin, periodic acid Schiff, or Gomori methenamine silver. After staining, histological examination was performed under light microscopy.

### Statistical Analysis

Categorical variables were compared between two groups using Fisher exact test. Continuous variables were compared among groups with equal variance by analysis of variance (ANOVA) followed by *post hoc* Tukey–Kramer HSD tests for pair-wise comparisons. If the standard deviations differed among groups, means were compared by Kruskal–Wallis test and *post hoc* Dunn’s test for pair-wise comparisons. Correlations between ocular fungal burden and cytokine/chemokine levels were evaluated by Pearson’s test. A *P*-value <0.05 (two tailed) was considered significant for all tests. All variables at *P* < 0.20 by univariate analysis were included in multivariate stepwise logistic regression analysis in epidemiological investigations. Statistical analyses in epidemiological studies were performed with EZR (Saitama Medical Center, Jichi Medical University), which is a graphical user interface for R (The R Foundation for Statistical Computing). More precisely, it is a modified version of R commander designed to add statistical functions frequently used in biostatistics (Kanda, 2013). Statistical analyses in animal studies were performed using GraphPad Prism, version 7 (GraphPad Software, La Jolla, CA, United States).

## Results

### Greater Incidence of Ocular Complications During *C*. *albicans* Infection Than Non-albicans *Candida* Infection

We retrospectively investigated the epidemiological data on *Candida* infection at Toranomon Hospital and identified 123 cases of candidemia during the study period, of which 99 were included in this study. Among these 99 patients, *C*. *parapsilosis* was the most common infective species, followed by *C*. *albicans* and *C*. *glabrata*. *C albicans* and *C*. *parapsilosis* co-infection was detected in one patient. Serum BDG was measured during the treatment period in 97 patients (98%).

Twenty of 99 candidemia patients were diagnosed with ocular candidiasis. The general clinicodemographic characteristics and *Candida* species distribution of the patients with ocular candidiasis and non-ocular candidiasis are summarized in **Tables 1**, **2**, respectively. The rate of *C*. *albicans* bloodstream infection was significantly higher in the ocular candidiasis group than the non-ocular candidiasis group (76% vs. 13%). In contrast, the rates of hematological malignancies, antifungal agent prophylaxis, and neutropenia were significantly higher in the non-ocular candidiasis group. Serum BDG values did not differ between the entire candidiasis cohort and the subgroup with *C*. *albicans* bloodstream infection (**Supplementary Figures S1A,B**). In addition, the proportion of patients seropositive for BDG values did not differ between ocular and non-ocular candidiasis groups (75% vs. 58%). Multivariate analysis showed that only *C*. *albicans* bloodstream infection was significantly associated with ocular candidiasis [Odds ratio 20.7, 95% CI (4.84–88.4), *P* < 0.0001], suggesting that ocular candidiasis is most likely to occur during *C*. *albicans* bloodstream infection regardless of underlying diseases or comorbidities.

**Table 1:** Epidemiological and medical characteristics of the patients in ocular candidiasis and non-ocular candidiasis groups.

Demographic or clinical characteristics	Ocular candidiasis (*N* = 20)	Non-ocular candidiasis (*N* = 79)	Univariate analysis *p*-value	Multivariate analysis *P*-value
Age, median years (range)	74 (41–89)	66.5 (20–93)	0.04	0.57
Male/Female	14 / 6	62 / 18	0.56	-
Fever, median °C (range)^∗^1	39.2 (36.0–40.1)	38.4 (35.5–40.7)	0.06	0.27
Septic shock	2 (10%)	12 (15%)	0.73	-
**Underlying malignancies**				
Hematological malignancies^∗^2	2 (10%)	31 (39%)	0.02	0.31
Solid organ malignancies	10 (50%)	28 (35%)	0.30	-
**Comorbidities**				
Diabetes mellitus	6 (30%)	15 (19%)	0.36	-
Liver cirrhosis	1 (5%)	1 (1%)	0.36	-
Chronic kidney diseases	4 (20%)	17 (22%)	>0.99	-
Abdominal surgery	2 (10%)	8 (10%)	>0.99	-
Central venous catheter insertion^∗^3	18 (90%)	69 (87%)	>0.99	-
**Immunosuppressive conditions**				
Neutropenia (<500/ μL)	0 (0%)	15 (19%)	0.04	>0.99
Corticosteroid usage	11 (55%)	41 (52%)	0.81	-
Other immunosuppressive agents	1 (5%)	18 (23%)	0.11	0.27
**Causative *Candida* species^∗^4**				
*C. albicans*	16 (80%)	10 (13%)	<0.01	<0.01
Non-albicans *Candida*	5 (25%)	69 (87%)		
Breakthrough candidemia	0 (0%)	27 (34%)	<0.01	0.99
**Breakdowns of antifungal agents**				
Micafungin	-	25 (93%)		
Liposomal amphotericin B	-	2 (7%)		
**Serum β-D-glucan evaluation**				-
BDG value (median, pg/mL)	49.3	20.5	0.14	-
Positive rate of serum BDG	15 (75%)	45 (58%)	0.20	0.16
The overall 30-day mortality rate	2 (10%)	18 (23%)	0.35	-

^∗^*1 Body temperature at the time of blood sampling; ^∗^2 Including acute or chronic leukemia (both myeloid and lymphoid), malignant lymphoma, and multiple myeloma; ^∗^3 Includes cervical, subclavian, femoral, and peripheral inserted central catheters; ^∗^4 *C. albicans* and *Candida parapsilosis* were simultaneously detected from blood culture in one patient with ocular candidiasis*.

**Table 2:** *Candida* species distribution in ocular candidiasis and non-ocular candidiasis groups.

*Candida* species	Ocular candidiasis (*N* = 20^∗^)	Non-ocular candidiasis (*N* = 79)
*Candida albicans*	16	10
Non-albicans *Candida*	5	69
**Breakdowns of non-albicans *Candida***		
*Candida parapsilosis*	2	39
*Candida glabrata*	2	17
*Candida tropicalis*	1	5
*Candida lusitaniae*		3
*Candida krusei*		2
*Candida africana*		1
*Candida fermentati*		1
*Candida guiiliermondii*		1

^∗^*Candida albicans and *C*. *parapsilosis* were simultaneously detected from blood culture in one patient with ocular candidiasis*.

### Greater Eye Invasion and Colonization by *Candida albicans* Than Non-albicans *Candida*

**FIGURE 1:**
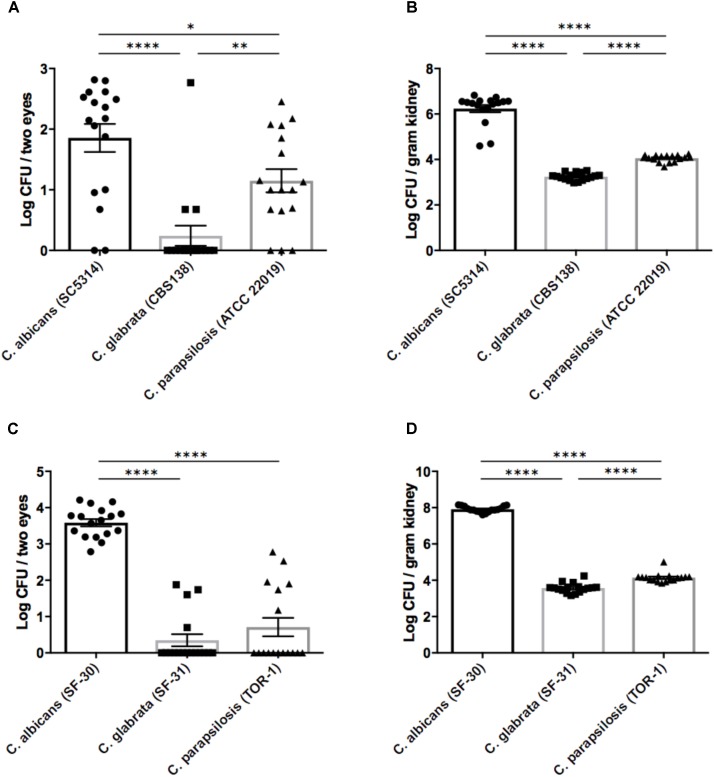
Organ fungal burden was significantly higher in mice during *Candida albicans* infection than non-albicans infection. **(A–D)** The fungal burden in the eyes and kidneys after injection of *Candida* species reference strains **(A,B)** or clinically isolated strains **(C,D)**. Kidney and ocular fungal burdens are shown as the Log CFU/g kidney and Log CFU/2 eyes, respectively. All results are expressed as mean ± standard error of the mean from three independent experiments with a total of 17 tissue samples per group. ^∗^*P* < 0.05, ^∗∗^*P* < 0.01, and ^∗∗∗∗^*P* < 0.0001.

On the basis of the above results, we performed *in vivo* experiments using our mouse ocular candidiasis model to compare the propensity for eye infection and severity of ocular inflammation among *Candida* species following bloodstream infection. Based on our epidemiological data and previous reports, *C*. *albicans*, *C*. *glabrata*, and *C*. *parapsilosis* are the most common *Candida* species causing in-hospital infection; accordingly, we decided to compare these species using both reference strains and clinical isolated strains. We injected reference strains of *C*. *albicans*, *C*. *glabrata*, and *C*. *parapsilosis* into separate mouse groups as described and then examined the fungal burdens in eyes and kidneys 3 days after injection. The fungal burden in eyes was significantly higher during *C*. *albicans* infection compared with non-albicans *Candida* infection (**Figure 1A**). In the kidneys as well, *C*. *albicans* burden was over 100-fold greater than non-albicans *Candida* burden (**Figure 1B**). Similar results were found using clinically isolated strains (**Figures 1C,D**).

**FIGURE 2:**
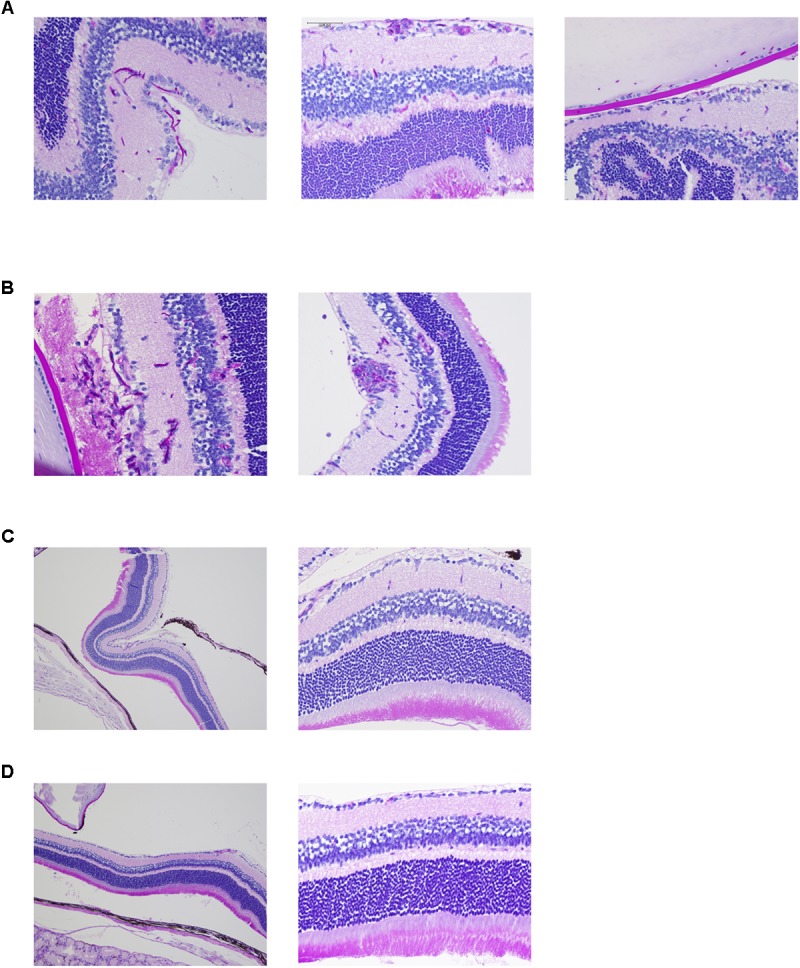
*Candida albicans* demonstrates greater invasive capacity into mouse retina than non-albicans *Candida.* **(A–D)** Histopathological analysis of mouse eyes infected by *C*. *albicans* reference strain **(A)**, *C*. *albicans* clinically isolated strain **(B)**, *Candida glabrata* clinically isolated strain (**C**, left panel showing lower magnification), and *Candida parapsilosis* clinically isolated strain (**D**, left panel showing lower magnification). These sections were stained with Periodic acid Schiff.

Ocular complications were compared among pathogens by histopathological analysis. Both the reference strain and clinically isolated strain of *C*. *albicans* invaded the mouse retina in the form of hyphae and also invaded the vitreous body in one sample (**Figures 2A,B** and **Supplementary Figures S2A,B**). In addition, accumulation of inflammatory cells around *C*. *albicans* was detected. Conversely, there were no signs of fungal invasion into the eye during infection by *C*. *glabrata* or *C*. *parapsilosis* (**Figures 2C,D** and **Supplementary Figures S2C,D**). These results suggest that *C*. *albicans* can more easily invade the retina and cause profound inflammation compared with non-albicans *Candida*, indicating that these species-specific propensities for ocular candidiasis are due to differences in invasive capacity.

### Elevation of Pro-inflammatory Mediators and Chemotactic Factors in Mouse Eyes During *C*. *albicans* Infection but Not Non-albicans *Candida* Infection

**FIGURE 3:**
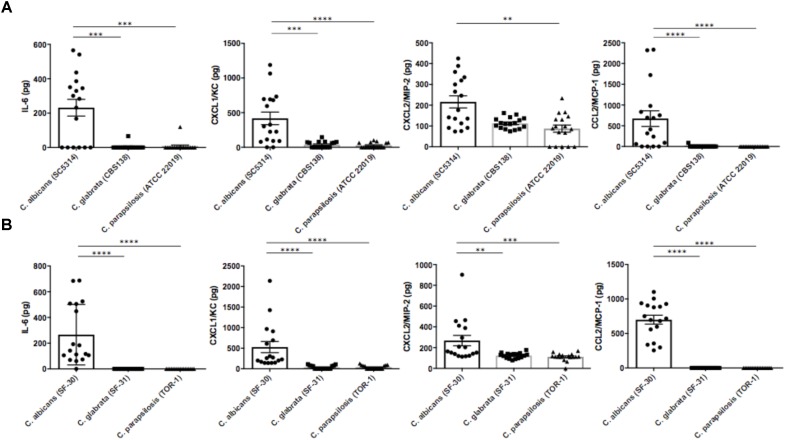
Interleukin-6 and neutrophil- or monocyte-attractive chemokines were highly induced in mouse eyes during *C*. *albicans* systemic infection. **(A,B)** Intraocular concentrations of IL-6, CXCL1/KC, CXCL2/MIP-2, and CCL2/MCP-1 3 days after injection of the *C*. *albicans* reference strain **(A)** or clinically isolated strain **(B)**. Ocular concentrations are expressed in pg/mouse. All results are expressed as mean ± standard error of three independent experiments with a total of 17 tissue samples per group (corresponding to **Figure 2A**). ns, not significant, ^∗∗^*P* < 0.01, and ^∗∗∗∗^*P* < 0.0001.

To identify factors inducing ocular inflammation following invasion, we measured several cytokines and chemokines in mouse eye homogenates by ELISA after *Candida* injection. Ocular concentrations of IL-6, CXCL1/KC, CXCL2/MIP-2, and CCL2/MCP-1 were significantly elevated during *C*. *albicans* infection compared with non-albicans *Candida* infection by the reference strains (**Figure 3A**). Surprisingly, IL-6 was not detected in most eyes following *C*. *parapsilosis* injection despite detection of this species in some eye samples. In contrast, the levels of IL-1β, IL-12p40, and TNF-α in ocular homogenate did not differ significantly among the three reference strain infection groups (**Supplementary Figure S3A**). In the preliminary experiment, these cytokines and chemokines were not elevated in uninfected mouse eyes (data not shown). Ocular concentrations of IL-6, CXCL1/KC, CXCL2/MIP-2, and CCL2/MCP-1 were also higher following injection of the *C*. *albicans* clinical strain compared with the other clinical strains (**Figure 3B**). In addition, ocular IL-1β was higher in mice injected with the clinical *C*. *albicans* strain than in mice injected with clinically isolated non-albicans *Candida*. Conversely, ocular TNF-α and IL-12p40 were significantly lower in mice with *C*. *albicans* infection compared with those with *C*. *parapsilosis* infection (**Supplementary Figure S3B**).

**FIGURE 4:**
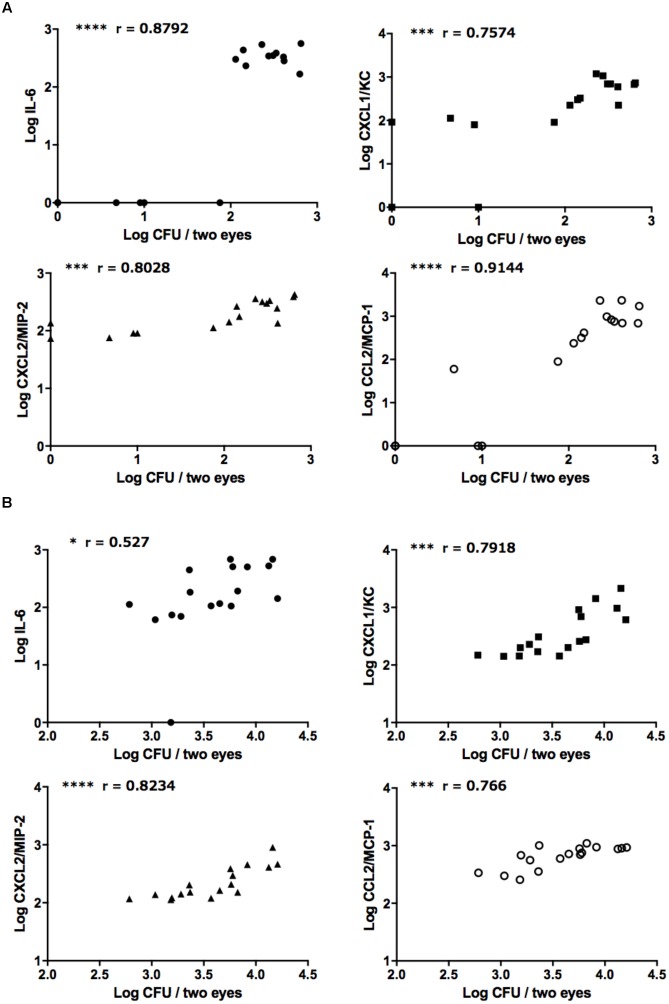
Strongly positive relationships between ocular *C*. *albicans* burden and ocular concentrations of IL-6 and neutrophil/monocyte-attractive chemokines. **(A,B)** Relationships between ocular *C*. *albicans* burden and IL-6, CXCL1/KC, CXCL2/MIP-2, and CCL2/MCP-1 following injection of the reference strains **(A)** or clinically isolated strains **(B)**. Ocular fungal burden is shown as Log CFU/2 eyes and ocular cytokine or chemokine levels as Log cytokine or chemokine (pg/mouse). The results of three independent experiments were pooled for analysis, each with 17 tissue samples. “r” is the Pearson’s correlation coefficient. ^∗^*P* < 0.05, ^∗∗∗^*P* < 0.001, and ^∗∗∗∗^ *P* < 0.0001.

Correlation analysis revealed strong positive correlations between ocular fungal burden and IL-6, CXCL1/KC, CXCL2/MIP-2, and CCL2/MCP-1 after injection of both the reference strain and clinically isolated strain of *C*. *albicans* (**Figures 4A,B**). On the other hand, there were no correlations between ocular *C*. *parapsilosis* fungal burden and the levels of these chemokines/cytokines (**Supplementary Figures S4A,B**). During infection by the *C*. *glabrata* reference strain, ocular IL-6 and MCP-1 were positively correlated with ocular fungal burden; however, the concentrations of these mediators were low and not detected in most infected mice, indicating only mild inflammation induced by *C*. *glabrata* (**Supplementary Figures 4C,D**). Consistent with the propensity of *C*. *albicans* for high ocular fungal burden, invading *C*. *albicans* induced severe inflammation by strongly stimulating the release of inflammatory cytokines and neutrophil or monocyte chemotactic factors. Conversely, non-albicans *Candida* did not exhibit the same invasive capacity or induce the release of inflammatory mediators, especially *C*. *parapsilosis*. These differences in the ocular inflammatory cytokine and chemokine profiles between *C*. *albicans* and non-albicans *Candida* likely explain the difference in ocular candidiasis frequency and severity following blood infection.

### Greater Inflammatory Cell Invasion Into Mouse Eyes During *C*. *albicans* Infection Than Non-albicans *Candida* Infection

**FIGURE 5:**
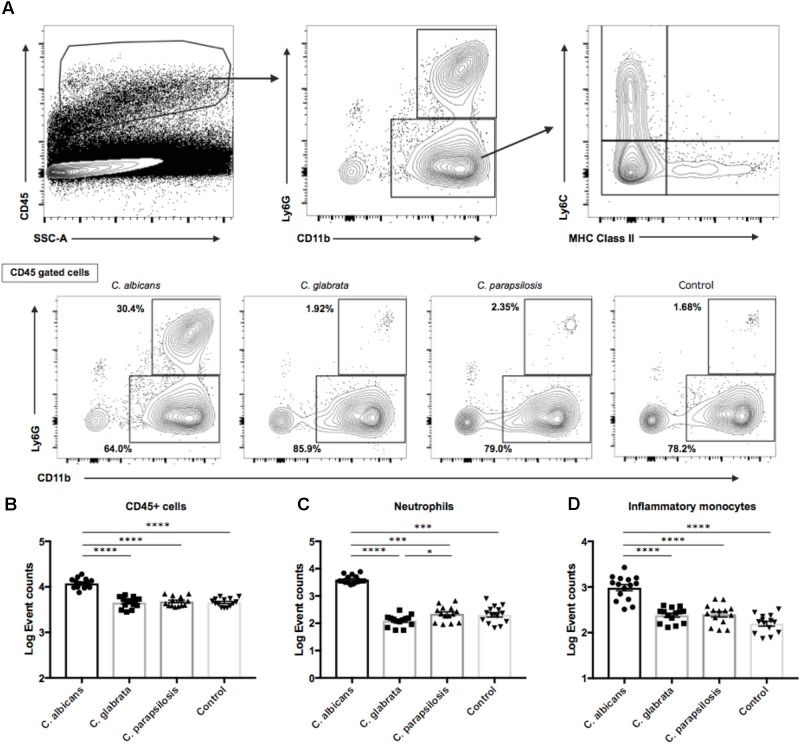
Neutrophil and inflammatory monocyte accumulations in mouse eyes were significantly greater during *C*. *albicans* infection than non-albicans *Candida* infection. **(A)** For flow cytometry analysis, gates were set for CD45 + cells. **(B–D)** Event counts of **(B)** ocular hematopoietic cells (CD45 +), **(C)** neutrophils (CD11b + Ly6G +), and **(D)** inflammatory monocytes (CD11b + Ly6G-Ly6C + MHC classII-). Cell numbers are shown as Log Event count. All results are expressed as mean ± standard error of the mean from three independent experiments including 15 *C*. *albicans*-infected samples and 14 samples for each non-albicans group. ^∗^*P* < 0.05, ^∗∗^*P* < 0.01, ^∗∗∗^*P* < 0.001, and ^∗∗∗∗^*P* < 0.0001.

On the basis of the above results, we examined the ocular inflammatory cell profile associated with each *Candida* species by flow cytometry. We infected mice with reference strains of each *Candida* species and quantified inflammatory cells in the eye after 3 days (**Figure 5A**). The number of CD45^+^ cells was higher in mice infected by *C*. *albicans* compared with non-albicans *Candida* infection; in fact, there were no differences in CD45^+^ cell numbers between the non-albicans *Candida* group and uninfected controls (**Figure 5B**). Among CD45^+^ cells, the numbers of ocular neutrophils and inflammatory monocytes were significantly elevated in mice infected by *C*. *albicans* compared with the non-albicans *Candida* group and uninfected controls (**Figures 5C,D**). In addition, the number of resident monocytes was also higher in mouse eyes during *C*. *albicans* infection (**Supplementary Figure S5A**). Conversely, the number of ocular macrophages was slightly lower in mice infected by *C*. *albicans* compared with non-albicans *Candida* and control groups (**Supplementary Figures S5B,C**). There was also a difference in neutrophil number between *C*. *parapsilosis* and *C*. *glabrata* groups, although this was quite small. Collectively, these results suggest that the greater invasive capacity of *C*. *albicans* induces a stronger inflammatory cytokine and chemokine response, leading to greater accumulation of neutrophils and inflammatory monocytes in the eye and more severe retinal inflammation (e.g., as manifested by white spots or focal infiltrates).

## Discussion

Although ocular candidiasis is a serious complication of candidemia, there are few previous reports describing the differences in ocular complications among *Candida* species. In our epidemiological investigation, *C*. *albicans* tended to cause ocular complications more frequently than non-albicans *Candida*, in accord with previous reports. Moreover, in our mouse ocular candidiasis model, the fungal burden, inflammatory cytokine and chemokine concentrations, and the numbers of ocular neutrophils and inflammatory monocytes were significantly higher following *C*. *albicans* injection compared with non-albicans *Candida* injections. These results indicate the stronger propensity for ocular candidiasis following *C*. *albicans* infection results from greater ocular invasive capacity and concomitantly greater secretion of inflammatory mediators and recruitment of neutrophils and inflammatory monocytes.

In the epidemiological study, *C*. *albicans* was the most frequent pathogen in ocular candidiasis, in accord with previous studies (Donahue et al., 1994; Shah et al., 2008; Oude Lashof et al., 2011; Lingappan et al., 2012; Nagao et al., 2012; Durand, 2013). In these previous reports, however, *C*. *albicans* was also the most frequently isolated species from blood cultures (Oude Lashof et al., 2011; Nagao et al., 2012). In contrast, *C*. *parapsilosis* was the most frequent pathogen in our investigation; nevertheless, the rate of ocular candidiasis caused by *C*. *albicans* was significantly higher than that caused by non-albicans *Candida*, suggesting that *C*. *albicans* has a stronger propensity for eye invasion, colonization, and induction of ocular candidiasis. In addition, serum BDG values were not significantly different between patients with ocular candidiasis and those without ocular candidiasis in our study, whereas previous reports concluded that serum BDG could be the marker of ocular candidiasis (Takebayashi et al., 2006; Nagao et al., 2012). One possible explanation for this discrepancy is the unique distribution of *Candida* species among sites. In our study, *C*. *parapsilosis* was the most frequent pathogen for candidemia, and non-albicans *Candida* accounted for approximately three quarters of all candidemia cases. The other possibility is the presence of antifungal prophylaxis. A previous report found that serum BDG was significantly lower in patients with breakthrough candidemia (Abe et al., 2014). Given our result that BDG seropositivity is not significantly higher in patients with ocular candidiasis, clinicians should not exclude the possibility of ocular candidiasis even if serum BDG is low.

In mouse experiments, *C*. *albicans* but not the other species test strongly induced inflammatory cytokine or neutrophil chemotactic chemokine release. Neutrophil accumulation was also found in the rabbit ocular candidiasis model, although the factors inducing neutrophil migration were not identified (Omuta et al., 2007). Our mouse experiments revealed that IL-6, KC/CXCL1, MIP-2/CXCL2, and MCP-1/CCL2 were strongly induced by *C*. *albicans* retinal invasion. On the other hand, other inflammatory cytokines, such as IL-12p40, IL-1β, or TNF-α, were not elevated; therefore, the aforementioned mediators appear to have central roles in *C*. *albicans*-induced ocular inflammation. Although there are no previous reports describing ocular candidiasis in mice, it was reported that KC/CXCL1, but not IL-6, impacts intraocular inflammation in mice during *Bacillus cereus* infection (Parkunan et al., 2016). In that report, neutrophil infiltration and inflammation as confirmed by histology were significantly less severe in CXCL1-/- mice compared with WT mice, indicating the importance of CXCL1 during *B*. *cereus* intraocular infection. On the other hand, another study reported a substantial role for IL-6 in the retinal inflammatory response in a mouse ocular toxoplasmosis reactivation model (Rochet et al., 2015). In our study, IL-6 and other chemokines also increased during *C*. *albicans* infection aside from neutrophil-activating KC/CXCL1. These chemokines and IL-6 may recruit other immune cells as flow cytometry revealed inflammatory monocyte infiltration into mouse eyes. Given our results and previous reports, IL-6, CXCL1, and other neutrophil or monocyte chemotactic factors likely all contribute to inflammation in ocular candidiasis.

There are several limitations to this study. First, the epidemiological study was retrospective and the number of cases involved was not large. However, most cases of candidemia were reviewed by experts in infectious diseases, so there were relatively few missing data on ocular candidiasis or serum BDG except for patients in terminal phases. Second, the variety of *Candida* species and the number of strains was limited to three in mouse experiments. Although many *Candida* species cause bloodstream infections, these are the top three according to previous studies and accounted for over 80% of candidemia cases in our epidemiological study (Oude Lashof et al., 2011; Nagao et al., 2012). Therefore, our findings are likely applicable to other institutions. Finally, our mouse ocular candidiasis model may reflect only the early phase of inflammation as we injected sublethal fungal burden to cause ocular candidiasis. Although the accumulations of neutrophils and inflammatory monocytes were apparent in the early phase and likely made major contributions to ocular inflammation, further investigation using other ocular candidiasis models are necessary to evaluate the late phase.

In summary, this is the first report describing differences in fungal burden, histopathological manifestations, chemotactic factor profiles, and invading inflammatory cell profiles among ocular candidiasis cases induced by *C*. *albicans* and non-albicans *Candida* in mice. Notably, results were in accord with epidemiological investigations in that *C*. *albicans* was both the most prevalent causative pathogen in humans and exhibited the greatest ocular invasive capacity in mice. Ocular candidiasis appeared more likely in cases of systemic infection by *C*. *albicans* compared with other *Candida* species, and the inflammation is more severe because of the greater cytokine/chemokine release and concomitant recruitment of neutrophils and monocytes compared with non-albicans *Candida* infection.

## Author Contributions

MA and YK designed the experiments. MA performed the experiments, acquired, and analyzed the data. KU, ST, SN, MiK, and YM contributed to the mouse experiments. SO, MuK, HA, and AY contributed to the epidemiological study. SS, MS, and KS contributed to the histopathological study. MA and YK drafted the manuscript. All the authors reviewed the manuscript.

## Conflict of Interest Statement

The authors declare that the research was conducted in the absence of any commercial or financial relationships that could be construed as a potential conflict of interest.
