# Supplementary material for: Differences in Ocular Complications Between Candida albicans and Non-albicans Candida Infection Analyzed by Epidemiology and a Mouse Ocular Candidiasis Model
Source: Front Microbiol. 2018 Oct 17;9:2477. doi: 10.3389/fmicb.2018.02477 (PMC6199462; doi:10.3389/fmicb.2018.02477)
Supplement: Supplementary file 1 [file Data_Sheet_1.docx]

Supplementary Material

**Differences in Ocular Complications Between *Candida albicans* and Non-albicans *Candida* Infection Analyzed by Epidemiology and a Mouse Ocular Candidiasis Model**

Masahiro Abe^*^, Yuki Kinjo^*^, Keigo Ueno, Shogo Takatsuka, Shigeki Nakamura, Sho Ogura, Muneyoshi Kimura, Hideki Araoka, Sota Sadamoto, Minoru Shinozaki, Kazutoshi Shibuya, Akiko Yoneyama, Mitsuo Kaku, and Yoshitsugu Miyazaki

***** Correspondence: Masahiro Abe, MD, and Yuki Kinjo, MD, PhD

Department of Chemotherapy and Mycoses, National Institute of Infectious Diseases, Tokyo, Japan

1-23-1 Toyama, Shinjuku-ku, Tokyo 162-8640, Japan

Tel: +81-3-5285-1111; Fax: +81-3-5285-1272

E-mail:To Masahiro Abe：[masa-a@niid.go.jp](mailto:masa-a@niid.go.jp), and To Yuki Kinjo：[ykinjo@niid.go.jp](mailto:ykinjo@niid.go.jp)

| Antigen | Clone | Supplier |
| --- | --- | --- |
| CD45 | 30-F11 | Biolegend |
| CD11b | M1/70 | Biolegend |
| CD11b | M1/70 | TONBO |
| Ly6C | HK1.4 | Biolegend |
| Ly6G | 1A8 | Biolegend |
| F4/80 | BM8 | Biolegend |
| I-A/I-E | M5/114.15.2 | Biolegend |
| CD11c | N418 | Biolegend |

**Supplementary Table: Antibodies used in this study**


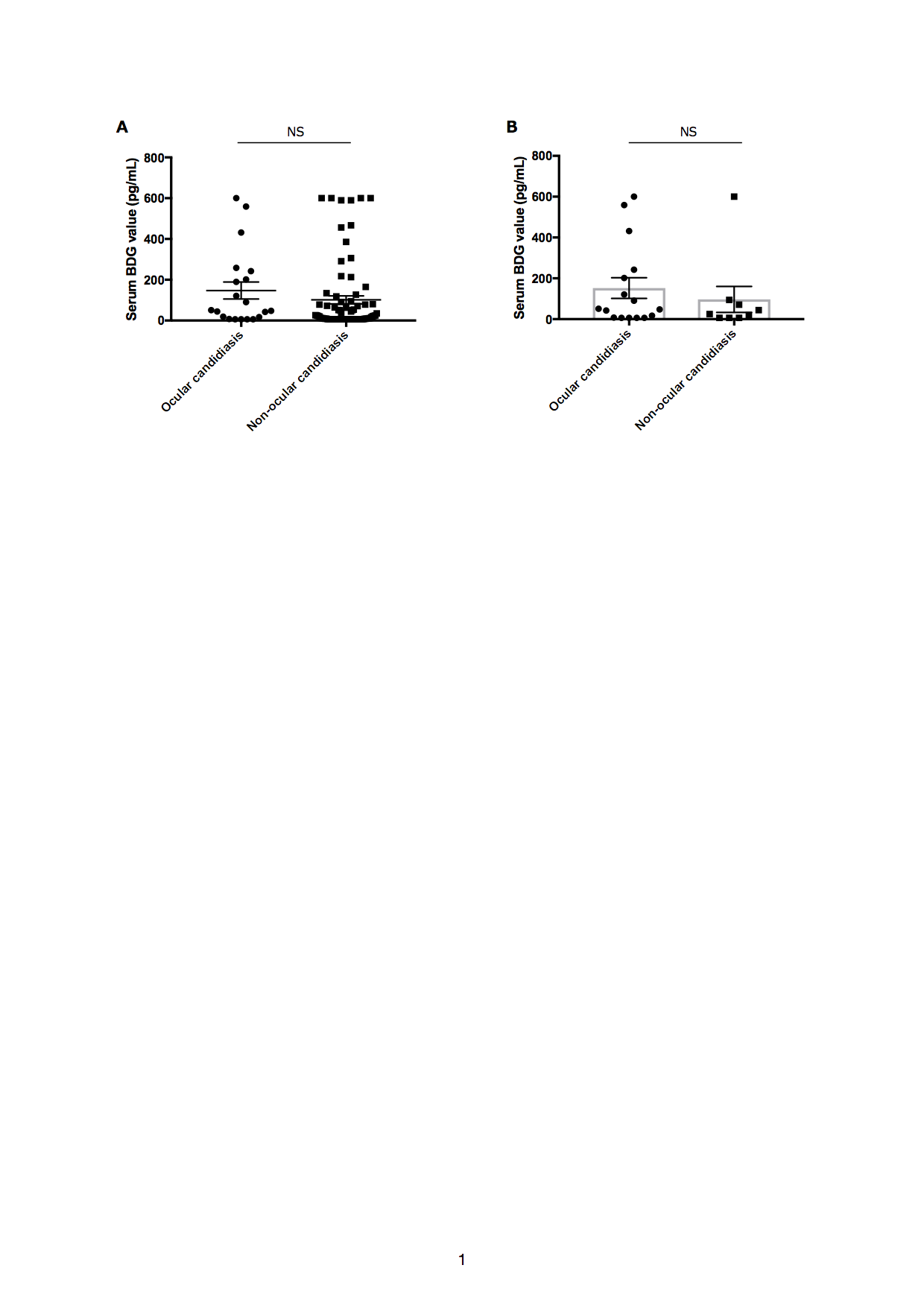
**Supplementary Figure 1. Serum β-D-glucan did not differ significantly between ocular candidiasis and non-ocular candidiasis groups.** (A, B) The serum BDG values of ocular candidiasis and non-ocular candidiasis groups including all *Candida* species (A) or just those with *C*.*albicans* bloodstream infection (B). All results are expressed as mean ± standard error of the mean. ns, not significant.


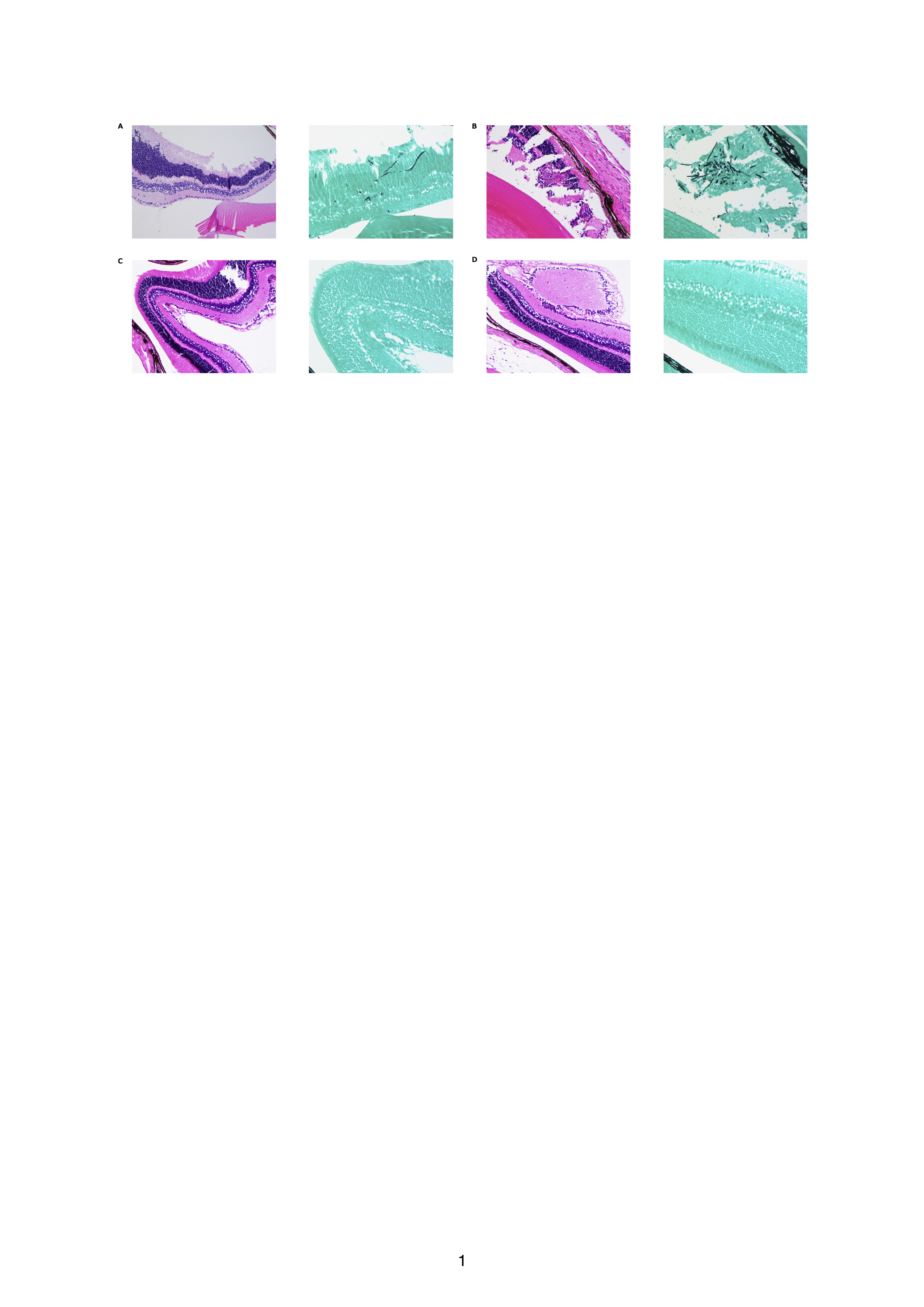
**Supplementary Figure 2.** (A−D) Histopathological analysis of mouse eyes infected by *C*.*albicans* reference strain (A), *C*.*albicans* clinically isolated strain (B), *C*.*glabrata* clinically isolated strain, and *C*.*parapsilosis* clinically isolated strain. Left panel of each slides shows middle magnification of Hematoxylin & eosin stain and right panel shows high magnification of Gomori methenamine silver stain.

**
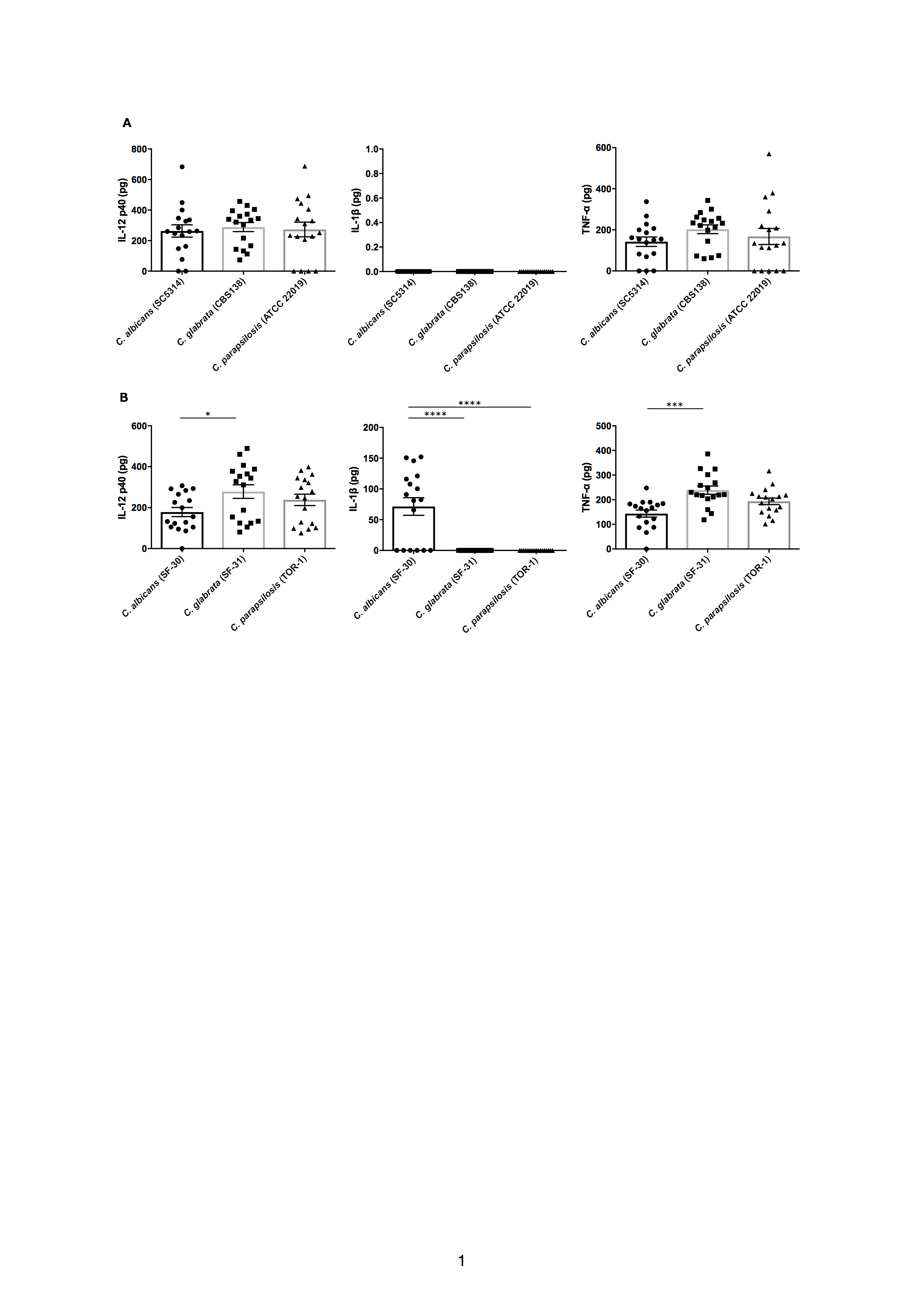
Supplementary Figure 3. Inflammatory cytokines were not abundantly induced by *C*.*albicans* infection.** (A, B) The values of ocular IL-12p40, IL-1β, and TNF-α 3 days after injection of the *C*.*albicans* strain (A) or clinically isolated strain (B). Ocular cytokine and chemokine levels are expressed in pg/mouse. All results are expressed as mean ± standard error of the mean from three independent experiments, each including 17 tissue samples per group (corresponding with Figure 2A). ns, not significant; * *P* < .05; *** *P*< .001; **** *P* < .0001.

**
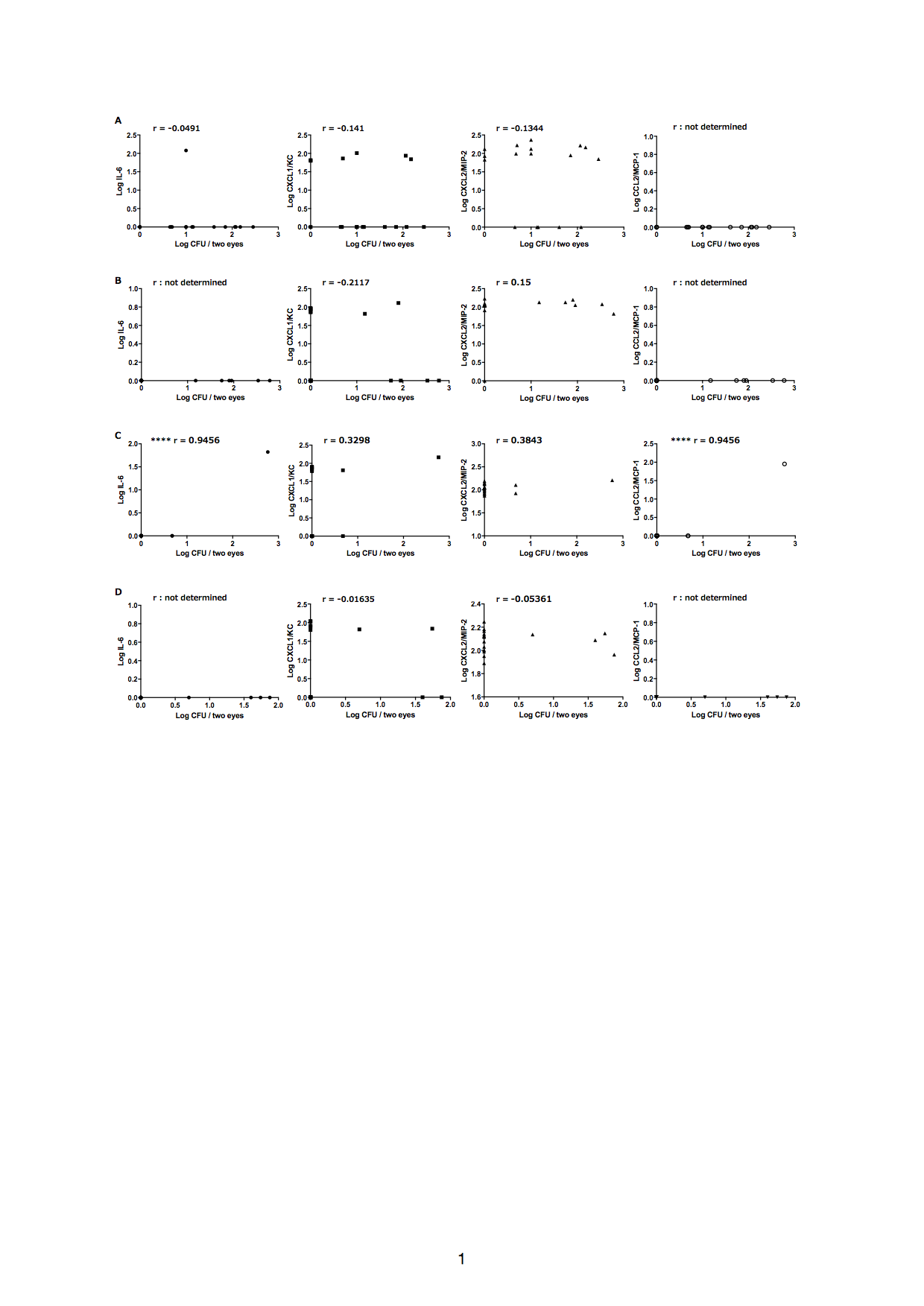
Supplementary Figure 4. There were few positive relationships between ocular non-albicans *Candida* burdens and most inflammatory cytokine or chemokine levels.** (A, B) Relationships between ocular *C*.*parapsilosis* burden and IL-6, CXCL1/KC, CXCL2/MIP-2, and CCL2/MCP-1 3 days after injection of the reference strain (A) or clinically isolated strain (B). (C, D) Relationships between ocular *C*.*glabrata* burden and these same cytokines or chemokines 3 days after injection of the reference strain (C) or clinically isolated strain (D). Ocular fungal burden is shown as Log CFU/2 eyes and ocular cytokine or chemokine level as Log cytokine or chemokine concentration in pg/mouse. The results of three independent experiments were pooled for analysis, each with 17 tissue samples. “r” is the Pearson’s correlation coefficient. **** *P* < .0001.

**
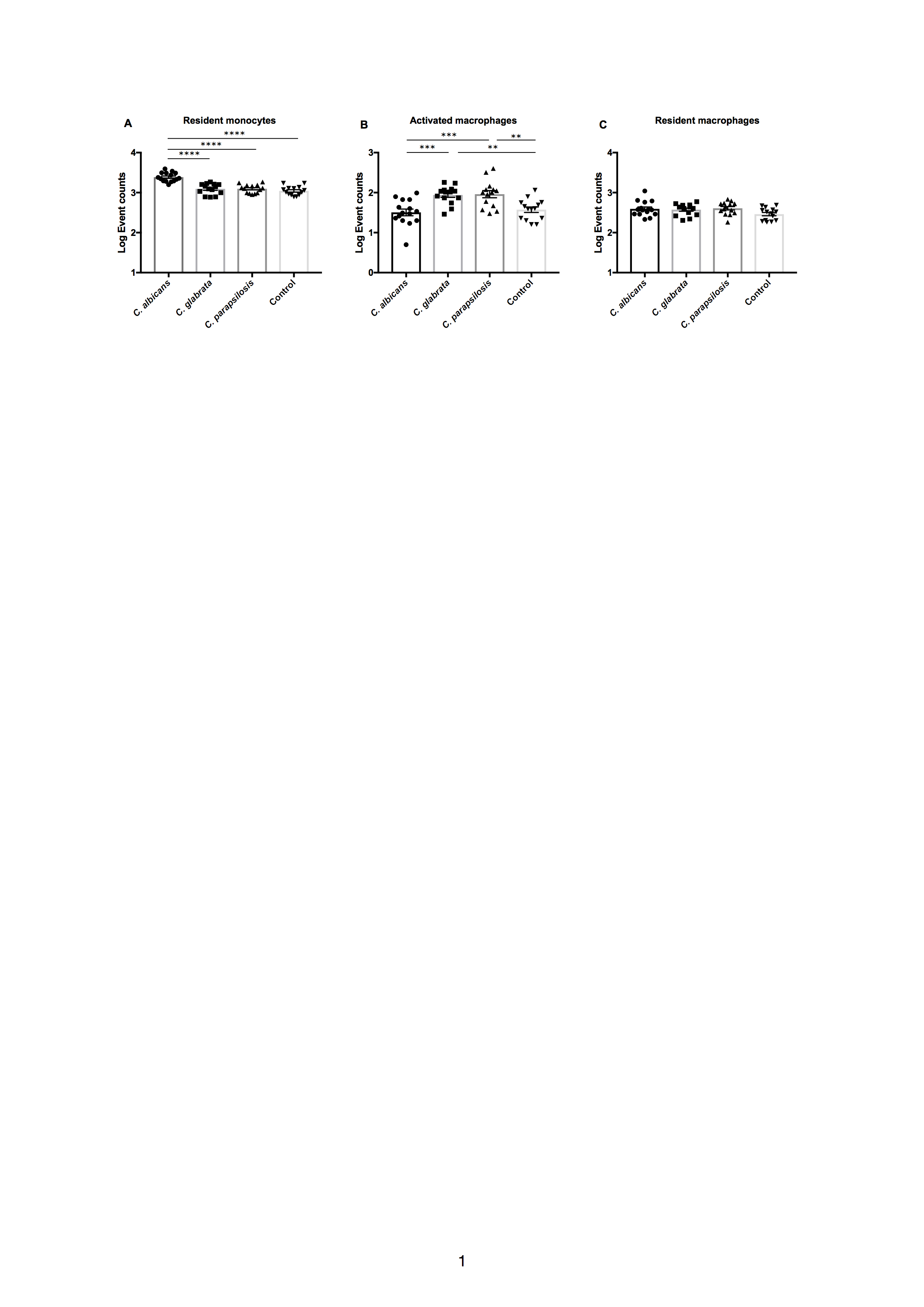
Supplementary Figure 5. Resident monocytes, but not macrophages, were significantly elevated in the mouse eye during *C*.*albicans* injection compared with non-albicans *Candida* infection.** (A−C) The event counts of (A) ocular resident monocytes (CD11b+ Ly6G− Ly6C− MHC classII−), (B) activated macrophages (CD11b+ Ly6G− Ly6C+ MHC classII+), and (C) resident macrophages (CD11b+ Ly6G− Ly6C− MHC classII+). Cell numbers are in Log Event counts. All results are expressed as mean ± standard error of the mean from three independent experiments including 15 tissue samples for the *C*.*albicans* group and 14 samples for each non-albicans group. ** *P* < .01, *** *P* < .001, **** *P* < .0001.
